# DMSO Solubility Assessment for Fragment-Based Screening

**DOI:** 10.3390/molecules26133950

**Published:** 2021-06-28

**Authors:** Shamkhal Baybekov, Gilles Marcou, Pascal Ramos, Olivier Saurel, Jean-Luc Galzi, Alexandre Varnek

**Affiliations:** 1 Laboratoire de Chémoinformatique UMR 7140 CNRS, Institut Le Bel, University of Strasbourg, 4 Rue Blaise Pascal, 67081 Strasbourg, France; sbaybekov@unistra.fr (S.B.); g.marcou@unistra.fr (G.M.); 2 Institut de Pharmacologie et de Biologie Structurale, Université de Toulouse CNRS, UPS, 205 Route de Narbonne, 31077 Toulouse, France; pascal.ramos@ipbs.fr (P.R.); olivier.saurel@ipbs.fr (O.S.); 3 Biotechnologie et Signalisation Cellulaire UMR 7242 CNRS, École Supérieure de Biotechnologie de Strasbourg, University of Strasbourg, 300 Boulevard Sébastien Brant, 67412 Illkirch, France; galzi@unistra.fr; 4 ChemBioFrance—Chimiothèque Nationale UAR3035, 8 Rue de L’école Normale, CEDEX 05, 34296 Montpellier, France

**Keywords:** DMSO solubility, QSPR, fragment-based screening, outlier detection, NMR

## Abstract

In this paper, we report comprehensive experimental and chemoinformatics analyses of the solubility of small organic molecules (“fragments”) in dimethyl sulfoxide (DMSO) in the context of their ability to be tested in screening experiments. Here, DMSO solubility of 939 fragments has been measured experimentally using an NMR technique. A Support Vector Classification model was built on the obtained data using the ISIDA fragment descriptors. The analysis revealed 34 outliers: experimental issues were retrospectively identified for 28 of them. The updated model performs well in 5-fold cross-validation (balanced accuracy = 0.78). The datasets are available on the Zenodo platform (DOI:10.5281/zenodo.4767511) and the model is available on the website of the Laboratory of Chemoinformatics.

## 1. Introduction

Screening methods have become indisputably an integral part of the drug discovery process [1,2], from hit identification to the evaluation of pharmacological properties. Over the past decades fragment-based screening (FBS) has gained a broad acceptance as an efficient alternative to the conventional high-throughput screening (HTS) [2,3]. This is related to the core idea of FBS, which involves analysis of relatively small libraries containing simple yet diverse organic scaffolds, or fragments, and the identification of hit fragments, that will be developed into more potent lead compounds. Among the basic requirements for fragment-like compounds, well covered by the “rule of three” guidelines [4,5], solubility issues require serious attention [6,7].

Low solubility directly affects the availability of a compound in solution, which may potentially lead to masking of its actual activity. This is notably important for compounds in FBS libraries since the typical concentration of samples is around 1 mM [8,9,10]. Such a relatively high concentration is related to the low binding affinity of fragments, usually found in the range of μM–mM [11]. The assessment of weak ligand–target interactions, requires highly sensitive techniques such as NMR spectroscopy, etc. One of the solvents commonly used in screening methods is dimethyl sulfoxide (DMSO), a well-established standard [12].

Due to the significance of this physicochemical property, the topic of solubility prediction has been and still remains relevant. The challenge of this subject is related to the complexity of the dissolution phenomenon, which is dictated by structural features, solid state, and other physicochemical properties [13]. Very few statistical models designed to predict DMSO solubility have been reported in the literature [12,14], with only one being publicly available [15]. Thus, Tetko et al. [15] reported a consensus model combining random forest, decision tree and Associative Neural Network individual models, trained on a large and structurally diverse dataset. However, the threshold used for categorizing compounds into “soluble” or “insoluble” classes was set to 10 mM, which is a common concentration of stock solutions.

**Figure 1:**
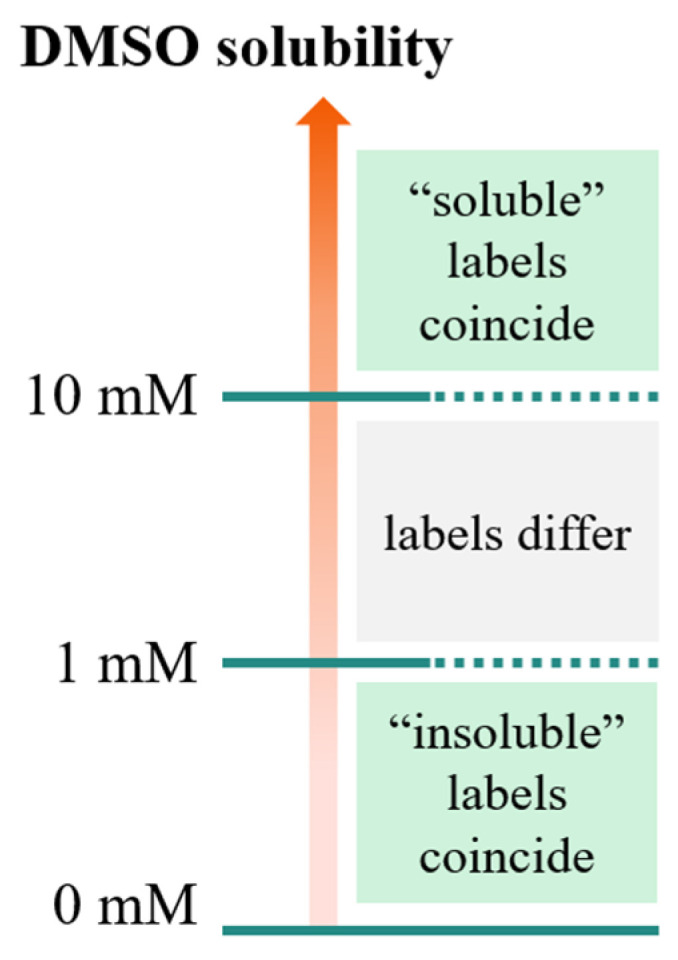
Solubility domains defined by the thresholds defined for stock solutions (10 mM) and FBS (1 mM). For these two threshold definitions, the “soluble”/“insoluble” labels coincide for solubility values larger than 10 mM and smaller than 1 mM, respectively. However, in the range 1–10 mM, molecules are considered soluble according to the FBS definition, but insoluble according to the stock solution definition.

As illustrated in Figure 1, compounds having a solubility in the range 1–10 mM, are considered soluble according to the FBS definition, but insoluble according to the stock solution definition. This means that the application of the “stock solutions” model by Tetko et al. [15] may lead to discarding compounds predicted as insoluble, but potentially suitable for FBS.

This motivated us to develop a classification model predicting fragment solubility in DMSO with a categorical threshold of 1 mM. The model was built on the experimental data provided by the “Plateforme Intégrée de Criblage de Toulouse” (PICT) screening platform. During the training stage, a set of erroneous measurements were identified and removed from the PICT set. The clean dataset was then used for building SVM models. With the help of a Generative Topographic Mapping (GTM) method, the PICT dataset was compared with fragment-like compounds from the Enamine database used for the preparation of the Tetko et al. [15] “stock solutions” model. This analysis revealed some structural motifs present uniquely in PICT. The datasets collected in this work are publicly available on the Zenodo platform (DOI:10.5281/zenodo.4767511). The consensus model is freely accessible on the website of the Laboratory of Chemoinformatics (http://infochim.u-strasbg.fr/cgi-bin/predictor2.cgi (accessed on 16 May 2021)).

## 2. Data

### 2.1. Experimental Protocol

In order to design a fragment library for NMR-based FBS, the stock solutions of 939 fragments were prepared at a final concentration of 100 mM in DMSO-d6, as described hereafter. The compounds, provided as powder, were dissolved at room temperature in DMSO-d6 under vigorous shaking until solubilization. Solutions were kept overnight at room temperature, then stored at −20 °C for months. The former solutions were then used for the preparation of a set of diluted solutions with a targeted concentration of 1 mM in DMSO-d6, to check by ^1^H NMR for each fragment the chemical structure conformity and the solubility. Stock solutions at 100 mM were thawed and kept overnight at room temperature before dilution and running the NMR analysis. NMR experiments were performed on a Bruker Avance III HD 600 MHz spectrometer (^1^H Larmor frequency) equipped with a cryoprobe. NMR experiments were performed with a 30° flip angle ^1^H pulse and 1.36 s of acquisition time (with a 20 ppm spectral width and a time domain 32 K complex of data points), and for each sample 32 scans were recorded with a repetition time delay of 5 s. NMR experiments were performed at 298 K and at atmospheric pressure. Quantification was performed with TopSpin, v. 3.5; Bruker Biospin software, by integration of the NMR peaks using the ERETIC2 [16] (Electronic Reference to access in vivo Concentrations) software based on the PULCON method [17]; an internal standard method which correlates the absolute intensities of spectra of compounds to be quantified with a reference spectrum. The reference spectrum was acquired as described above from a 1 mM isoleucine solution in DMSO-d6. The experimental error of solubility determination was estimated as 50 μM.

### 2.2. Data Description

The PICT dataset contained structures of 939 compounds with their corresponding DMSO concentration values ranging from 0 to 1000 μM. Since the expected concentration for DMSO samples was 1 mM, a threshold for making a division between soluble and insoluble categories was set to 1000 μM. Therefore, if concentration values were equal to 1000 μM it would be classified as soluble, and insoluble if the value was below the given threshold. Experimental error on the concentration was estimated at 50 μM; therefore, it was decided to remove a segment of the dataset in the range 900–999 μM, as in this range the soluble/insoluble label is ambiguous. After the removal of data points with missing solubility values and the aforementioned “gray area” zone, the number of compounds in the training set was reduced to 822, where 686 and 136 compounds belonged to “soluble” and “insoluble” classes, respectively. The key physicochemical parameters varied across the PICT set in the following ranges: calculated logP −3.8 – +3.94, molecular weight 150–302 Da, the number of hydrogen bond acceptors 0–6, and the number hydrogen bond donors 0–3.

### 2.3. Data Curation

The chemical structures were standardized using a ChemAxon Standardizer [18]. Applied rules included the removal of solvents, ions, explicitly indicated hydrogen atoms, neutralization, and aromatization. All stereo labels were skipped. A detailed description of the standardization protocol is provided in Supporting Information (“Standardization protocol” section). Erroneous measurements were then detected with the help of the outlier identification procedure (see below).

### 2.4. Filtered Enamine Data

A subset of the fragment-like compounds was extracted from the Enamine dataset used for training of the Tetko et al. model [15] with the help of a filter, matching the same ranges of variation as the PICT dataset for ClogP, molecular weight, number of H-donors and H-acceptors. The filtering resulted in the selection of 8314 fragment-like compounds out of the initial set of 50,620 compounds.

## 3. Method

### 3.1. Molecular Descriptors

ISIDA substructural molecular fragments (SMF) [19] were used in this study. SMF descriptors are derived solely from hydrogen suppressed 2D chemical graphs. They represent fragments of different topologies (sequences of atoms and bonds, sequences of atoms only, atom-centered fragments, triplets) and size (see Table S1 in Supporting Information). The minimal length of fragments varied between 2 and 3, whereas the maximal length varied between 2 and 8. Encoding of a given sequence by its terminal atoms (“atom pairs”) was also considered. A fragment occurrence is a descriptor value. Variation of the descriptors topology; type of sequence (explicit atoms or atom pairs and size) led to the generation of the pool of 182 subsets of descriptors. ISIDA descriptors were used in numerous QSAR studies [20,21,22].

### 3.2. Machine Learning Method

Classification models were built using the Support Vector Machine (SVM) machine learning (ML) algorithm. It was used for the selection of optimal descriptor sets, outlier identification and the generation of predictive models. The Libsvm 3.24 package [23] was used for the generation of linear SVM models. The Golden section search method was used in order to find the optimal cost parameter ranging from 0.01 to 1000 with a stopping criterion of 0.1. Optimization was performed to maximize 5-fold cross-validation (5-CV) balanced accuracy (BA).

### 3.3. Modeling Workflow

**Figure 2:**
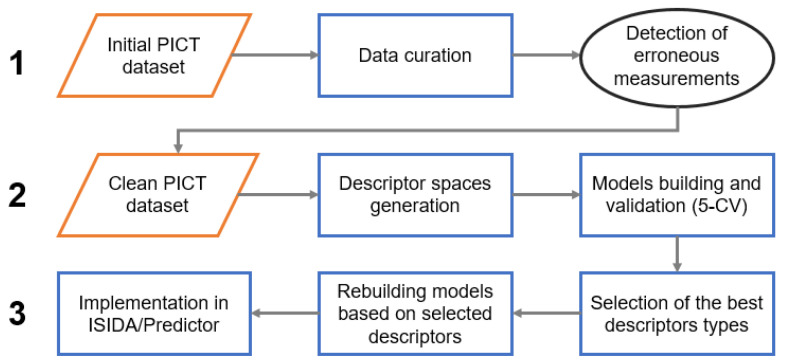
The modeling workflow.

The modeling workflow consisted of three main stages: (1) detection of erroneous measurements, (2) selection of relevant descriptor spaces and (3) model building and implementation (Figure 2). Detection of erroneous measurements was performed following a protocol from Ruggiu et al. [24] adapted in this study to classification tasks. This approach suggests the preparation of several individual models and the identification of the common badly predicted instances. For the curated PICT dataset, 26 various fragment descriptor spaces were generated. Each subset of descriptors was used for the modeling. Five models providing the best performance in 5-fold cross-validation were selected. At the next step, common false positives and false negatives (“outliers”) detected by all selected models at the training stage were identified and inspected by the experimental team. A vast majority of them were associated with technical problems and discarded from the dataset (see “Results and discussion” section). The resulting “clean” dataset was used in a new round of model building and validation.

At the next stage, 182 descriptor spaces were generated and used for the building and validation of SVM models. Models performing with BA ≥ 0.75 in 5-CV were selected; the highest BA = 0.80 was achieved for the model based on the atom centered fragments connecting atoms pairs derived for the sequences of atoms and bonds of 3–4 atoms length (type “IIAB(3-4)_R-P”, see Table S1 in Supporting Information). Descriptors involved in the selected models were then used to develop classification models on the entire “clean” PICT dataset. Obtained in such a way, 45 individual models formed a consensus model integrated into the ISIDA Predictor tool [25]. For any new molecule, the tool assigns a solubility label according to the majority of votes for the individual models. The predictive performance of the consensus model is reasonable (BA = 0.78 in 5-CV). Notice that the ISIDA Predictor accounts for the fragment control [26] applicability domain (AD) of each individual model. If a new molecule is outside of the AD, the model is not applied. Along with the predicted label, the tool provides a confidence estimation based on the ratio of the percentage models and prediction consistency.

The consensus model is freely available on the website of the Laboratory of Chemoinformatics (http://infochim.u-strasbg.fr/cgi-bin/predictor2.cgi (accessed on 16 May 2021)). In order to access the model, select the “PhysProp” option in the “general kind of property” section and then choose “Solubility DMSO” option from the “property to model” drop-down list. A user is invited to draw a molecule of interest or upload an SD file containing several compounds. Some screenshots illustrating the functioning of the ISIDA Predictor are given in the Supporting Information (Figure S5).

### 3.4. Generative Topographic Mapping

Generative Topographic Mapping (GTM) [27,28,29,30] is a dimensionality reduction method, which transforms a high-dimensional molecular descriptor space into a 2D latent space (“map”). This is achieved by introducing a 2D manifold into the high-dimensional space and adjusting a normal probability density, centered on the nodes of a rectangular grid superposed with the manifold, to the observed data distribution. Once the manifold is fitted, the compounds are projected on this 2D surface. GTM is widely used for the chemical space visualization, analysis, and compounds’ profiling [31].

Two maps were constructed: (i) for the PICT dataset and (ii) for the merged PICT and Enamine datasets. The method hyperparameters and type of fragment descriptors were optimized by maximizing the classes separation (“soluble/insoluble” for the PICT dataset and “PICT/Enamine” for the merged dataset). The compounds were encoded by atom centered fragments, including a given atom and atoms and bonds of its either 3 or 5 coordination spheres for the merged dataset and the PICT dataset, respectively. The data distribution was visualized using “class landscapes” [30], highlighting areas populated by soluble and insoluble compounds.

## 4. Results and Discussion

### 4.1. Data Visualization and Analysis

**Figure 3:**
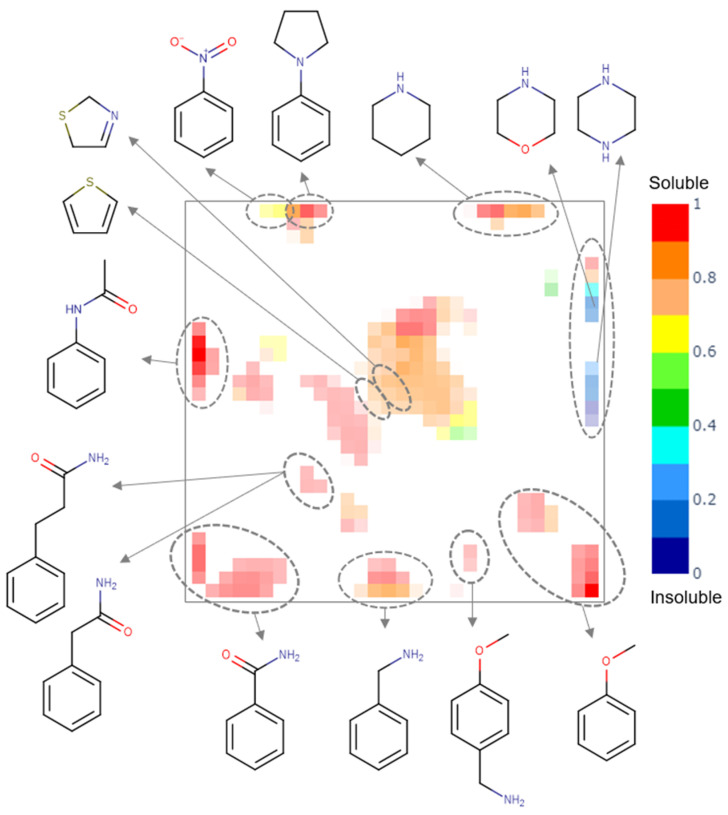
The class landscape for the PICT dataset. Blue and red zones are populated by insoluble and soluble molecules, respectively. Green and yellow zones contain a mixture of soluble and insoluble compounds.

A generative topographic map built for the PICT dataset shows several clusters populated by compounds of a particular chemotype (see Figure 3). Insoluble compounds bear piperazine and morpholine fragments, soluble compounds are mostly aromatic amines, amides, piperidines and ethers, whereas compounds bearing nitro-benzene, thiophene and dihydro-thiazole fragments can be either soluble or insoluble.

**Figure 4:**
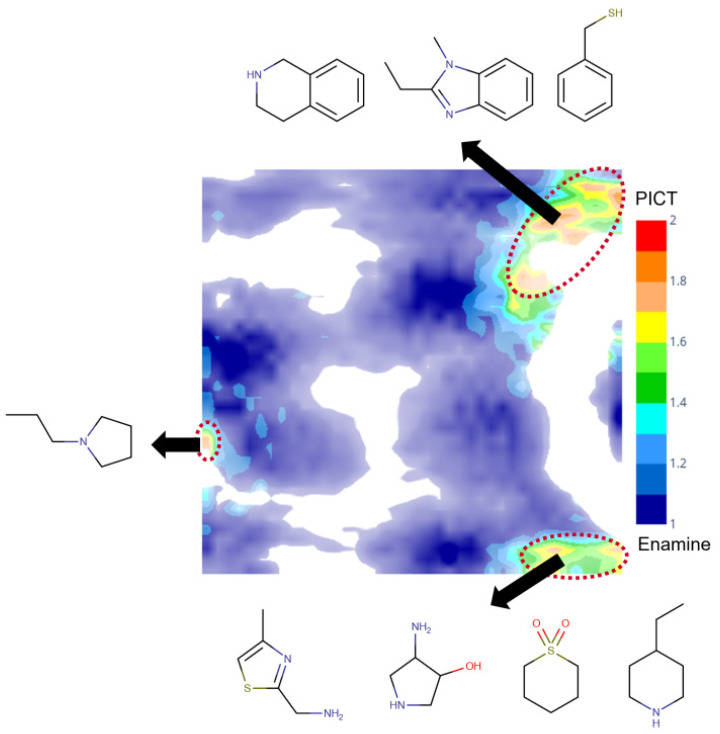
The class landscape depicting the coverage of a fragment-like chemical space by PICT and Enamine datasets. Blue and red zones are populated, respectively, by Enamine and PICT molecules. Green and yellow zones contain a mixture of compounds from the two datasets.

A comparative analysis of the PICT and filtered Enamine datasets was performed using a generative topographic map combining both datasets. Figure 4 shows a class landscape in which the color code characterizes the presence of Enamine or PICT compounds in a particular zone of the chemical space. The map well separates blue and red zones populated by Enamine and PICT compounds, respectively, which confirms the structural diversity of the two datasets. Detailed analysis of the red zones, reveal some particular structural motifs present in the PICT and absent in the Enamine dataset (Figure 4).

### 4.2. Erroneous Measurements Detection

As explained above, the outliers are compounds in which the predicted labels systematically do not match the experimental ones for none of the initially developed models. There are 34 outliers which belong to three categories: experimental errors, chemical instability, and unexplained discrepancies. The list includes 31 insoluble compounds predicted as soluble and three soluble molecules predicted as insoluble (see Table S3 in Supporting Information). These modeling results were reported to the PICT team for the reassessment of experimental values. The analysis showed that 15 out of 34 potential outliers resulted from a human error during the sample preparation. Overall, during the revision of the NMR spectra, nine compounds were found to have degradation signs, whereas the values of 19 samples were likely affected by experimental errors. These 28 confirmed outliers were discarded. The remaining six compounds were claimed to have no experimental issues. Some incorrectly predicted compounds and their correctly predicted close analogues form some sort of “solubility cliffs” (Table 1). Thus, compounds **1a** and **1b** differ by a methylene bridge between two cyclic fragments; the difference between compounds **2a** and **2b** results from the type of substituent (OH or CH_2_-OH) and its position in the piperidine ring, whereas compound **3b** has two methyl groups more than the compound **3a**. These cliffs are intriguing and require further structure-activity relationship (SAR) exploration, which is beyond the scope of this work.

### 4.3. “Stock Solutions” vs. FBS Models

For the sake of comparison, the “stock solutions” model by Tetko et al. [15] was applied to the PICT dataset and, vice versa, the FBS model was applied to the Enamine dataset. Only 87.4% of the Enamine data were found inside the applicability domain of at least one FBS individual model. On the other hand, 98.6% of the PICT dataset was covered by the AD of the “stock solution” model.

Results given in Table 2 show that both models predicted soluble compounds with a high accuracy, but failed to predict insoluble ones. The latter is not surprising when the FBS model is applied to the Enamine dataset: since solubility assignment thresholds of FBS and stock solution models differ, the compounds with a solubility in the range 1–10 mM are considered soluble according to FBS and insoluble according to stock solution models. On the other hand, the compounds in which the solubility value is smaller than 1 mM are considered insoluble according to both models. This could be explained by the fact that the PICT dataset contains some unique structural motifs, e.g., thiazole, benzimidazole or tetrahydroisoquinoline (see Figure 4). It also looks like these models (at least, the “stock solution” one) are biased toward the training set composition containing mostly soluble compounds.

## 5. Conclusions

This work combines experimental and chemoinformatics studies of the solubility of small molecules (“fragments”) in DMSO in the context of their application in fragment-based screening. Experimentally measured data (PICT dataset) were used for the development of the first classification model for DMSO solubility fragments (FBS model). Unlike the earlier reported “stock solution” model with the categorical threshold “soluble/insoluble” of 10 mM, our model uses a more suitable threshold for fragments of 1 mM. The model displays a reasonable predictive performance in 5-fold cross-validation (BA = 0.78). Both the experimentally measured data and developed model are freely available for users.

We have demonstrated that the developed model can efficiently be used to detect erroneously measured data. Among the 28 picked compounds pointed to by the model, nine compounds were found to have degradation signs, whereas the values of 19 samples were likely affected by experimental errors.

The comparison of the PICT and Enamine datasets performed with the help of a Generative Topographic Mapping approach showed that the PICT dataset contains some unique structural motifs absent in the Enamine collection.

The results reported here demonstrate a synergism between experimental and chemoinformatics teams for obtaining, analyzing and modeling of the DMSO solubility of small molecules (“fragments”) in the context of their application in fragment-based screening.

## Figures and Tables

**Table 1:** Example of incorrectly predicted compounds and their correctly predicted close analogues.

Incorrectly Predicted Compounds				Correctly Predicted Similar Compounds			
#	Compound structure	Exp	Pred	#	Compound structure	Exp	Pred
**1a**	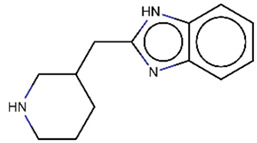	Soluble	Insoluble	**1b**	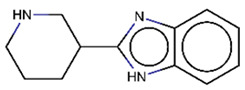	Insoluble	Insoluble
**2a**	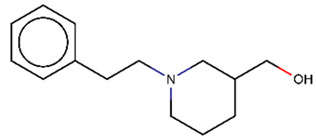	Soluble	Insoluble	**2b**	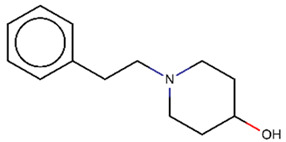	Insoluble	Insoluble
**3a**	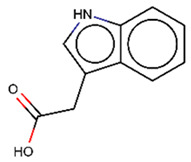	Insoluble	Soluble	**3b**	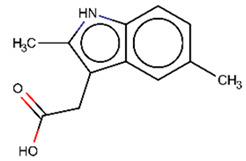	Soluble	Soluble

**Table 2:** Predictive performance of the FBS model on the filtered Enamine data, and of the “stock solution” model on the PICT data. The number of correctly predicted compounds with respect to the total number of compounds is given between the parentheses.

	FBS Model on Enamine Dataset	«Stock Solution» Model on PICT Dataset
Recall (soluble)	0.954 (6828/7156)	1 (676/676)
Recall (insoluble)	0.052 (6/115)	0.01 (1/101)

## Data Availability

The data presented in this study are available on the Zenodo platform (DOI:10.5281/zenodo.4767511) and in Supplementary Materials.

## Supplementary Materials

The following are available online: description of standardization rules, description of ISIDA fragment descriptors, description of statistical metrics, a list of models constituting the FBS consensus model, description of GTM parameters of class landscapes, a summary of predictions made on the “gray area” compounds, the outlier detection and removal workflow, a list of outliers, a list of reported classification models for the prediction of DMSO solubility and the screenshots showing the usage of the “Predictor” web-application containing our model, the PICT dataset containing experimental solubility values and class labels and the filtered Enamine dataset.

## Sample Availability

Samples of the compounds are not available.
