# Supplementary material for: DMSO Solubility Assessment for Fragment-Based Screening
Source: Molecules. 2021 Jun 28;26(13):3950. doi: 10.3390/molecules26133950 (PMC8271413; doi:10.3390/molecules26133950)
Supplement: Supplementary file 1 [file molecules-26-03950-s001.zip › molecules-1268375-supplementary.pdf]

Supplementary Materials of "DMSO Solubility Assessment for Fragment-Based Screening"

Shamkhal Baybekov [1], Gilles Marcou [1], Pascal Ramos [2], Olivier Saurel [2], Jean-Luc Galzi [3,4], Alexandre Varnek [1,]*

[1] Laboratoire de Chémoinformatique UMR 7140 CNRS, Institut Le Bel, University of Strasbourg, 4 Rue Blaise Pascal, 67081 Strasbourg, France

[2] Institut de Pharmacologie et de Biologie Structurale, Université de Toulouse CNRS, UPS, 205 route de Narbonne, 31077 Toulouse, France

[3] Biotechnologie et Signalisation Cellulaire UMR 7242 CNRS, École Supérieure de Biotechnologie de Strasbourg, University of Strasbourg, 300 Boulevard Sébastien Brant, 67412 Illkirch, France

[4] ChemBioFrance—Chimiothèque Nationale UAR3035, 8 Rue de l'École Normale, CEDEX 05, 34296 Montpellier, France

Table of Contents

Standardization protocol S2
Description of ISIDA Fragment Descriptors S4
Statistical metrics S4
List of individual models selected for the consensus S5
GTM parameters of class landscapes S6
Application of FBS model to the extracted "gray area" compounds S6
Outlier analysis S7
References S15

Standardization Protocol

We used “ChemAxon Standardizer” software[1] to standardize the compounds and the rules applied to this set are listed below, according to their order:

1. Clear Stereo—clears all stereo effects.
2. Remove Solvents—removes water molecules.
3. Remove Fragment—keeps a molecule with the largest number of atoms.
4. Remove Explicit Hydrogens—removes all explicitly indicated hydrogen atoms.
5. Neutralize—changes the charge of a specie to 0 by adjusting it (e.g. adding or removing hydrogens).
6. Transform thiazolopyrimidine—specific transformation rule.
7. Transform pyridinone—specific transformation rule.
8. Dearomatize—converts aromatic ring to Kekule representation.
9. Aromatize—converts Kekule representation to aromatic ring form. “Basic style aromatization” was chosen.

Steps 6 and 7 were created specifically for this data set, in order to convert particular compounds. The structure of these compounds as well as the transformation mechanism are shown in **Figure S1** and **Figure S2**. These additional rules were necessary since their absence yielded structurally less preferrable forms.

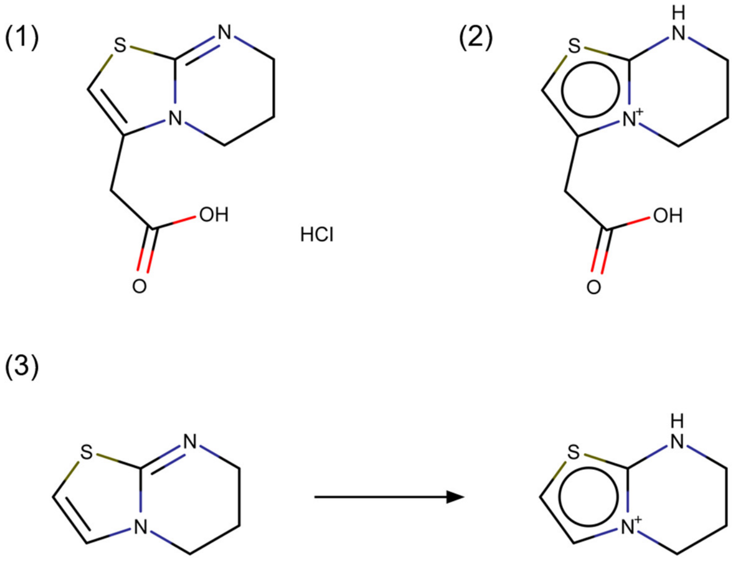

Figure S1. “Transform thiazolopyrimidine” standardization rule. Transformation **3** was applied to compound **1** to convert it to the form **2**.

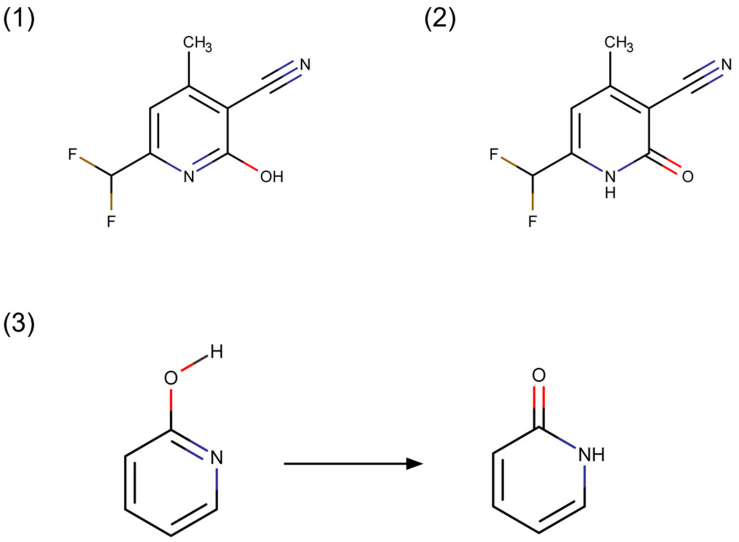

Figure S2."Transform pyridinone” standardization rule. Transformation **3** was applied to compound **1** to convert it to the form **2**.

Description of ISIDA Fragment Descriptors

In **Table S1**, a list of fragmentation types with their notations that were used in this study is provided. The minimal length of fragments was set either to 2 or 3, whereas the maximal length of fragments varied between 2 and 8. Atom count fragments were additionally calculated and mixed with every fragmentation type. "Atom pairs" was used as an additional option during the fragmentation. When "atom pairs" option is added, it removes all constitutional details of a sequence and gives only the number of constitutive atoms.

Table S1. Description of fragmentation types used in this study and their notations.

Fragmentation Type	Notation
Sequences of atoms only	IA
Sequences of atoms and bonds	IAB
Atom centered fragments based on sequences of atoms	IIA
Atom centered fragments based on sequences of atoms and bonds	IIAB
Atom centered fragments based on sequences of atoms of fixed length	IIA_R
Atom centered fragments based on sequences of atoms and bonds of fixed length	IIAB_R
Triplets	IIIA

Statistical Metrics

In our work, we mentioned two statistical metrics: balanced accuracy (BA) and recall. They are calculated by equations given below (TP – true positive; TN – true negative; FP – false positive; FN – false negative).

$$Recall(soluble) = \frac{TP}{TP + FN} \qquad Recall\ (insoluble) = \frac{TN}{TN + FP}$$

$$BA = \frac{Recall\ (soluble) + Recall\ (insoluble)}{2}$$

List of Individual Models Selected for the Consensus

In **Table S2**, a list of models that were used for preparing the consensus model is provided. All models were built using support vector machine (SVM) algorithm with linear kernel (Libsvm 3.24 package[2]). Golden section search method was used to find optimal cost parameters. Mean balanced accuracies (BA) is the average of each fold model's BA in 5-fold cross-validation.

Table S2. List of SVM models constituting the consensus model. Information about used descriptor spaces, mean BA over 5-fold and standard deviation is given. "P" stands for "Atom Pairs" fragmentation option. Other notations are described in the previous section.

Descriptor Space	Mean BA	Standard Deviation	Descriptor Space	Mean BA	Standard Deviation
IIAB(3-4)_R-P	0.802	0.059	IIAB(2-5)_R-P	0.764	0.075
IIAB(3-3)	0.800	0.064	IIA(2-4)	0.764	0.062
IIAB(3-3)_R	0.800	0.064	IIA(3-6)_R	0.761	0.069
IIAB(2-4)_R-P	0.793	0.062	IIA(2-4)_P	0.760	0.064
IIAB(2-4)_R	0.792	0.063	IIAB(2-5)_R	0.758	0.086
IIA(2-5)_R	0.789	0.071	IIA(3-8)_R	0.758	0.067
IIA(2-4)_R	0.780	0.064	IIAB(3-4)_P	0.758	0.058
IIAB(3-4)_R	0.780	0.062	IIAB(3-6)_R	0.757	0.072
IIAB(3-4)_R-P	0.780	0.070	IIAB(3-3)_R-P	0.757	0.042
IIA(3-5)_R	0.779	0.071	IIAB(3-3)_P	0.757	0.042
IIAB(2-3)_R	0.779	0.047	IIA(3-7)_R	0.757	0.081
IIA(3-4)_R	0.778	0.056	IIAB(3-6)_R-P	0.756	0.090
IIA(2-7)_R	0.775	0.070	IIA(3-3)	0.755	0.032
IIA(2-8)_R	0.774	0.072	IIA(3-3)_R	0.755	0.032
IIAB(2-6)_R-P	0.772	0.070	IIA(2-3)_R	0.755	0.069
IIAB(2-3)	0.772	0.057	IIAB(2-4)_P	0.755	0.067
IIIA(2-4)	0.772	0.051	IIAB(2-4)	0.755	0.070
IIA(2-6)_R	0.770	0.064	IIA(2-3)	0.754	0.073
IIA(3-4)	0.767	0.040	IIA(2-8)_R-P	0.753	0.069
IIA(2-6)_R-P	0.766	0.055	IIAB(2-8)_R-P	0.753	0.086
IIAB(2-3)_P	0.766	0.055	IIA(3-8)_R-P	0.751	0.098
IIAB(3-5)_R	0.765	0.082	IIAB(2-7)_R-P	0.750	0.072
IIAB(3-4)	0.765	0.052			

GTM Parameters of Class Landscapes

The class landscape presented in **Figure 4** was built on ISIDA atom centered fragment descriptors (IIAB(3-3)_R) with atom counts. The map was built using 40 RBF centers and has dimension of 40x40 nodes. The class landscape provided **Figure 3** was built on ISIDA fragment descriptors (IIAB(2-5)_R) with atom counts using 14 RBF centers, and the dimensions of the map are 30x30 nodes. BA obtained over 3 iterations of 3-fold cross-validation was 0.75.

Application of FBS Model to the Extracted "Gray Area" Compounds

As described in the "Data description" of the main paper, the "gray area", [900, 999] µM, was removed from the training set. FBS model was applied to this dataset and the summary of predictions is given in **Figure S3**. There were 103 compounds in the "gray area". FBS model predicted 83 compounds as soluble and 20 compounds as insoluble.

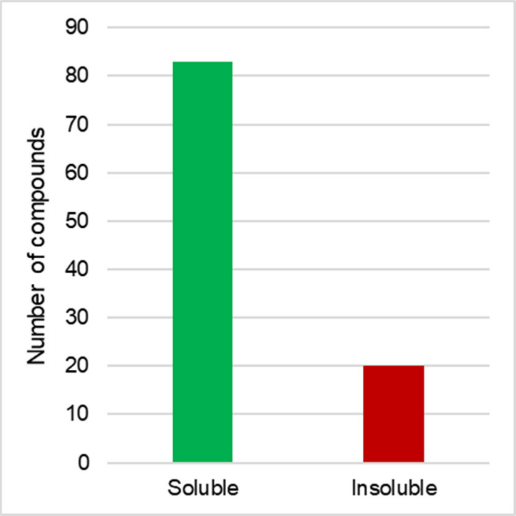

Figure S3. Summary of predictions made on the "gray area" compounds (103 compounds). 83 compounds were predicted as soluble, and 20 compounds were predicted as insoluble.

Outlier Analysis

In this section, the outlier detection workflow (**Figure S4**) and 34 outliers (false positives and negatives) are provided along with their similar analogues predicted correctly (**Table S3**). In comment cells, brief results of reassessment are given. Overall, there are 9 degraded, 19 erroneously prepared molecules and 6 measurements, where no experimental error was observed. Values given in "Exp" column are experimental solubility values in μM.

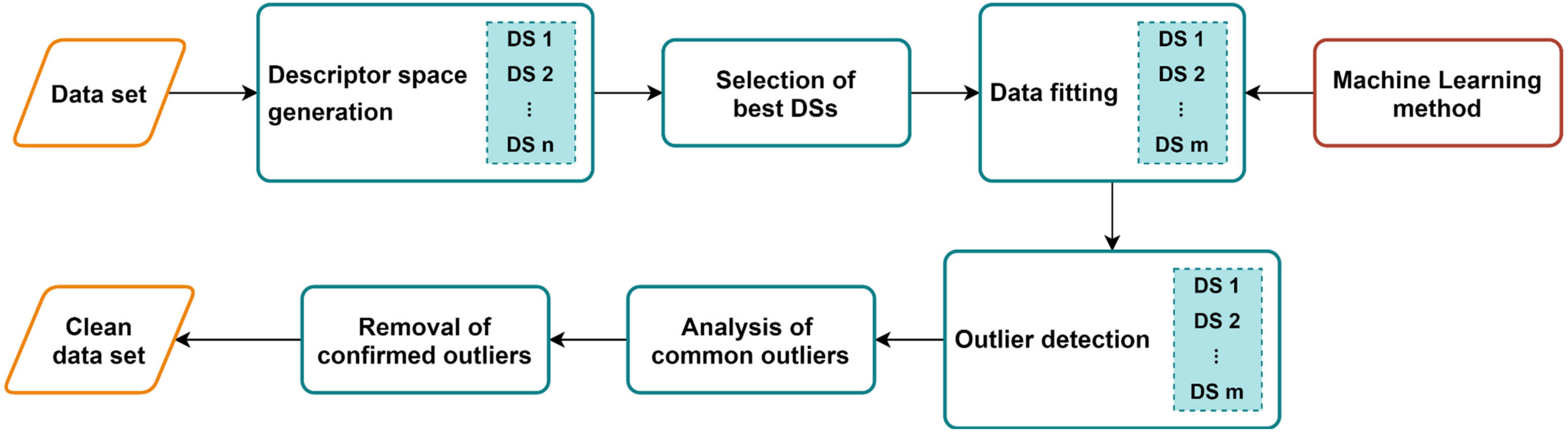

Figure S4. Outlier detection and removal workflow. "DS" stands for a descriptor space.

Table S3. The list of outliers and correctly predicted similar compounds. “Exp” column contains experimental solubility in µM and class label. “Pred” column contains class label assigned by the model. “Comment” section provides a brief information about the result of reassessment.

Incorrectly Predicted Compound					Correctly Predicted Similar Compound			
**#**	**Compound Structure**	**Exp**	**Pred**	**Comment**	**#**	**Compound Structure**	**Exp**	**Pred**
1a	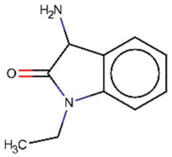	800 Insoluble	Soluble	Degraded	1b	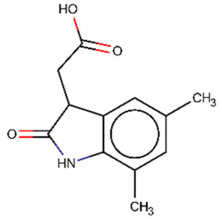	1000 Soluble	Soluble
2a	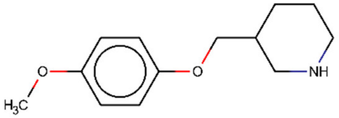	860 Insoluble	Soluble	Degraded	2b	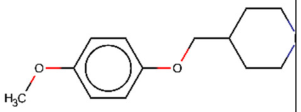	1000 Soluble	Soluble
3a	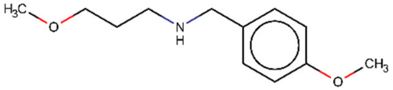	880 Insoluble	Soluble	Degraded	3b	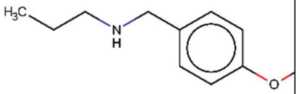	1000 Soluble	Soluble
4a	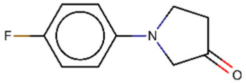	400 Insoluble	Soluble	Degraded	4b	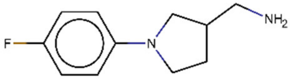	1000 Soluble	Soluble
5a	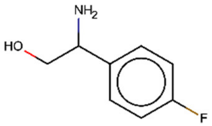	800 Insoluble	Soluble	Degraded	5b	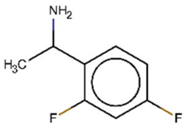	1000 Soluble	Soluble

6a	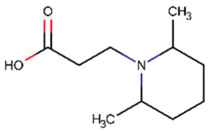	600<br>Insoluble	Soluble	Degraded	6b	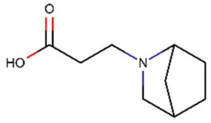	1000<br>Soluble	Soluble
7a	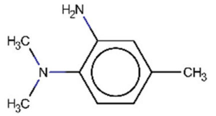	650<br>Insoluble	Soluble	Degraded	7b	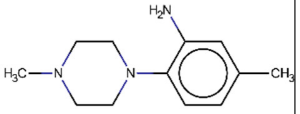	1000<br>Soluble	Soluble
8a	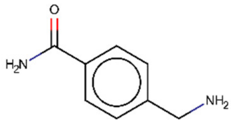	830<br>Insoluble	Soluble	Degraded	8b	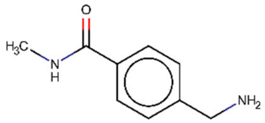	1000<br>Soluble	Soluble
9a	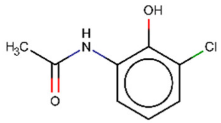	870<br>Insoluble	Soluble	Degraded	9b	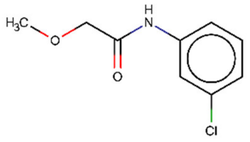	1000<br>Soluble	Soluble
10a	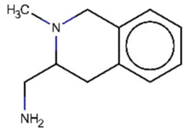	670<br>Insoluble	Soluble	Preparation error	10b	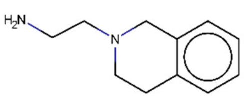	1000<br>Soluble	Soluble
11a	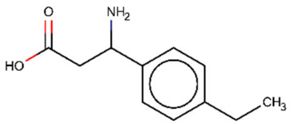	260<br>Insoluble	Soluble	Preparation error	11b	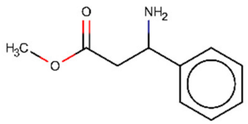	1000<br>Soluble	Soluble
12a	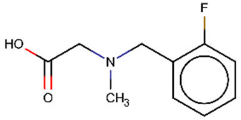	800<br>Insoluble	Soluble	Preparation error	12b	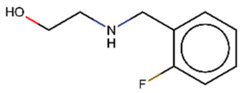	1000<br>Soluble	Soluble

13a	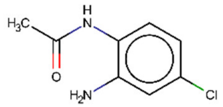	850<br>Insoluble	Soluble	Preparation error	13b	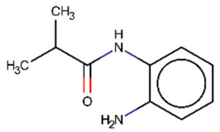	1000<br>Soluble	Soluble
14a	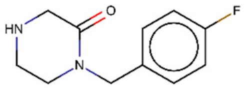	870<br>Insoluble	Soluble	Preparation error	14b	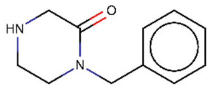	1000<br>Soluble	Soluble
15a	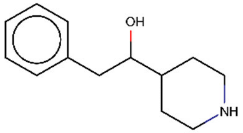	880<br>Insoluble	Soluble	Preparation error	15b	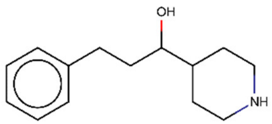	1000<br>Soluble	Soluble
16a	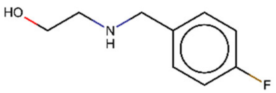	880<br>Insoluble	Soluble	Preparation error	16b	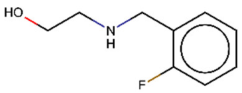	1000<br>Soluble	Soluble
17a	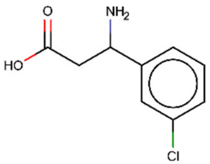	600<br>Insoluble	Soluble	Preparation error	17b	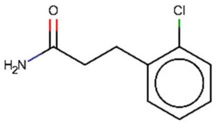	1000<br>Soluble	Soluble
18a	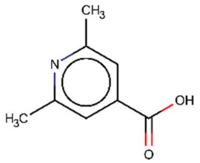	850<br>Insoluble	Soluble	Preparation error	18b	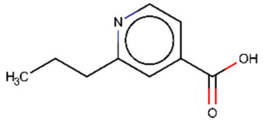	1000<br>Soluble	Soluble

19a	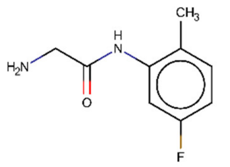	860 Insoluble	Soluble	Preparation error	19b	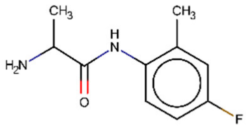	1000 Soluble	Soluble
20a	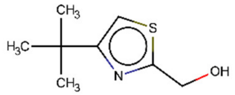	860 Insoluble	Soluble	Preparation error	20b	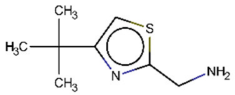	1000 Soluble	Soluble
21a	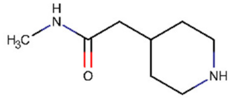	860 Insoluble	Soluble	Preparation error	21b	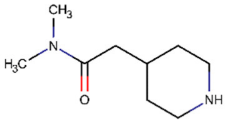	1000 Soluble	Soluble
22a	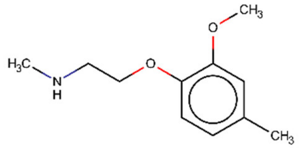	830 Insoluble	Soluble	Preparation error	22b	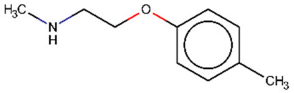	1000 Soluble	Soluble
23a	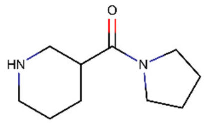	800 Insoluble	Soluble	Preparation error	23b	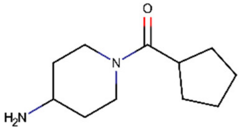	1000 Soluble	Soluble
24a	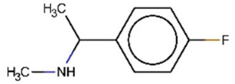	830 Insoluble	Soluble	Preparation error	24b	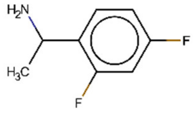	1000 Soluble	Soluble
25a	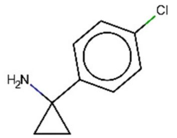	800 Insoluble	Soluble	Preparation error	25b	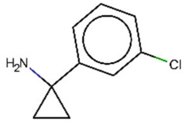	1000 Soluble	Soluble

26a	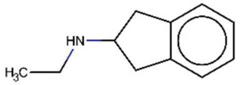	800<br>Insoluble	Soluble	Preparation error	26b	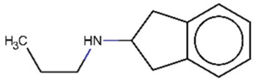	1000<br>Soluble	Soluble
27a	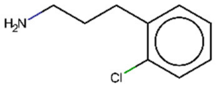	800<br>Insoluble	Soluble	Preparation error	27b	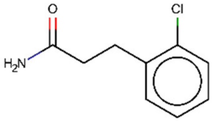	1000<br>Soluble	Soluble
28a	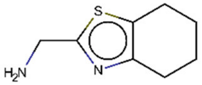	800<br>Insoluble	Soluble	Masked CH_2 signal	28b	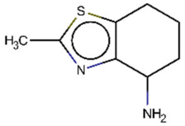	1000<br>Soluble	Soluble
29a	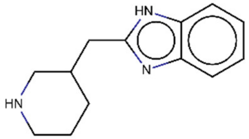	1000<br>Soluble	Insolubl<br>e	No experiment al error	29b	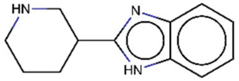	730<br>Insoluble	Insoluble
30a	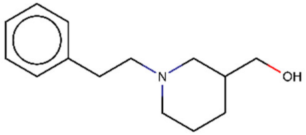	1000<br>Soluble	Insolubl<br>e	No experiment al error	30b	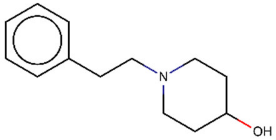	830<br>Insoluble	Insoluble
31a	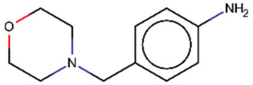	1000<br>Soluble	Insolubl<br>e	No experiment al error	31b	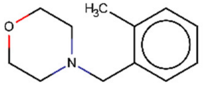	800<br>Insoluble	Insoluble
32a	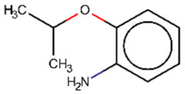	800<br>Insoluble	Soluble	No experiment al error	32b	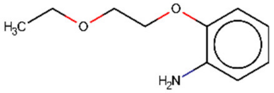	1000<br>Soluble	Soluble

33a	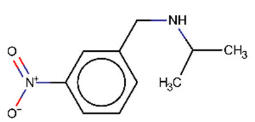	730 Insoluble	Soluble	No experimental error	33b	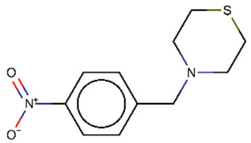	830 Insoluble	Insoluble
34a	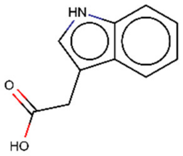	630 Insoluble	Soluble	No experimental error	34b	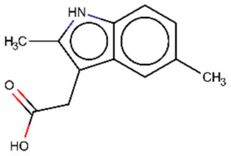	1000 Soluble	Soluble

Table S4. Reported classification models for prediction of DMSO solubility. Only the best models are listed in the table.

Authors	Dataset Size	Method	Descriptors	BA	Correctly Classified
Tetko et al.[15]	50620	consensus	physicochemical, constitutional, topological, geometric, electronic	0.87	NA
Balakin et al.[12]	65500	SOM[a]	physicochemical, constitutional, topological, geometric	NA	93%

[a] Kohonen self-organizing map

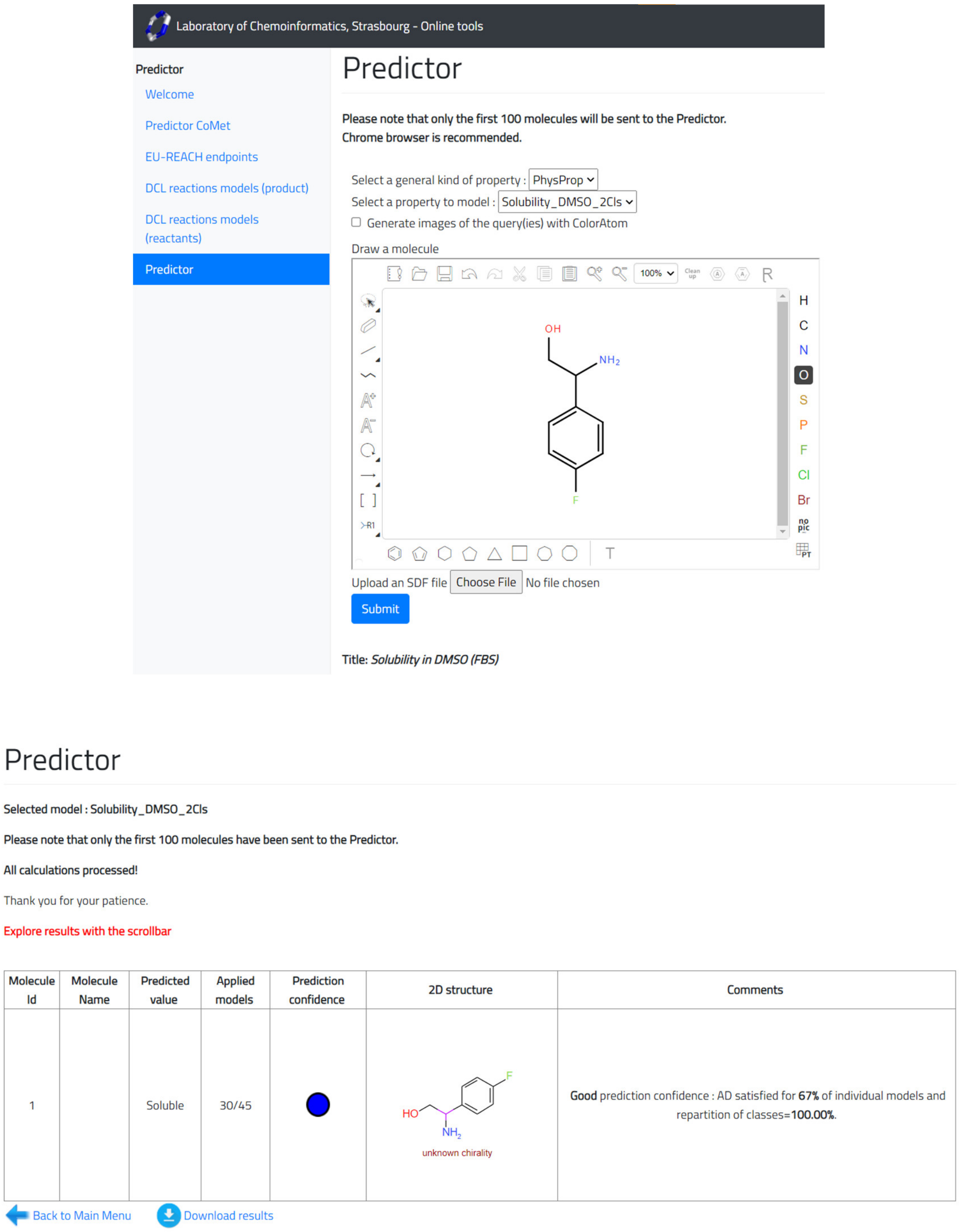

Figure S5. Screenshots displaying an example of "Predictor" web application usage.

References

1. ChemAxon Standardizer. Available online: http://www.chemaxon.com/.
2. Chang, C.-C.; Lin, C.-J. {LIBSVM}: A Library for Support Vector Machines. *ACM Trans. Intell. Syst. Technol.* **2011**, *2*, 27:1–27:27.
